# The Possibilities of Porcelain as a Filling Material

**Published:** 1902-08-15

**Authors:** W. T. Reeves


THE
DENTAL REGISTER.

Vol. LVI. August 15, 1902. No. 8.

### THE POSSIBILITIES OF PORCELAIN AS A FILLING MATERIAL.

BY W. T. REEVES, D.D.S.

Read before the Michigan Dental Association, June 10, 1902.

In presenting this subject for your consideration, I shall endeavor to show some of the possibilities of porcelain as a filling material, and when and where it can be used, and how it should be manipulated; also some theories of mine as to why it is the most permanent of all the materials we use to-day for the restoring of lost tooth structure.

*When and where it can be used.* Porcelain as a filling material has been used for many years, but it is within the past five years that it has been advanced to the position which it holds to-day. To you who are working along these lines I hope to bring some new principles that will stimulate you to a more extensive use of porcelain as a filling material. To such as are looking on from afar, waiting for the experimental stage to pass, I want to say, the time is now here, porcelain inlays are no longer an experiment, they are a proven success and have become a permanent feature in all dental operations.

Conservatism is commendable in all but when anything is a proven success it is time for all to ''get into the band wagon.''

When the ability and skill of the operator to successfully complete any given case, and the patient is able to recompense the operator for time and skill expended, then porcelain is the ideal filling material. I say ability, advisedly, for there is no work we do that requires such careful attention to every detail as the making of a porcelain inlay; the slighting or hurrying over of any portion of the work will result in failure. The old saying ''Practice makes perfect,'' applies with great force to this branch of our work. Any one of you would laugh at the student in your office, who may by observation have acquired a certain amount of knowledge, who should attempt to fill for a patient any of those cavities that looked so easy as he saw you do the work; you all know the amount of hard work you did before you acquired the skill in handling gold that makes the filling of difficult cavities, to-day, easier than the simple ones were when you began. This applies with double force to the making of porcelain inlays.

The person who in paper or discussion (and there have been a good many who have gone on record in the past few years) says ''that porcelain inlays are limited to the simpler cavities, easy of access, in the anterior teeth,'' occupies the same position as your student would if he should say that gold could only be put in simple cavities easy of access, because his inexperience limits the use of gold to such cavities in his hands. As you acquire experience you will go from easy to more difficult cavities until you become so proficient that the most extensive cavities in molar or bicuspid will be possible in your hands. Then you will restore with inlays teeth that it would have been impossible to fill with gold, and do it with the minimum amount of mental and physical strain upon both yourself and patient. There is no work that you do, from start to finish, that is so easy for the patient as the preparation of a cavity for the insertion of a porcelain inlay.

*Where porcelain can be put.* You will conclude from the foregoing that I place hardly any limitation as to where you can use porcelain as a filling material; it can be used in more places and with a greater degree of success than gold. I will briefly enumerate some of the persons for whom, and places where porcelain can be successfully used.

Those high-strung nervous temperaments for whom it is almost a physical impossibility to prepare a cavity even for a cement filling, to say nothing of gold, you can prepare the cavity for an inlay with comparative comfort, and when set you have done permanent work.

For the young in whose mouths gold fillings fail faster than cement wash out you will come very near doing permanent work with porcelain inlays.

For those refined, sensitive natures, that the extensive display of gold in the front of the mouth is an ever conscious annoyance, you can confer a great benefit, and restore to full contour and usefulness the worst broken down teeth, so that persons at close conversational range would not observe that artificial means had been resorted to.

For the aged, whose strength would not permit of the protracted operation of inserting a gold filling, an inlay can be made; for if necessary the work can be divided into two or three sittings of comparative short duration.

For those bordering on nervous prostration you can do permanent work with the minimum amount of nervous taxation.

For women during gestation, and for the young mother whose maternal duties make it impossible to take the time necessary for a large gold filling, in both of these cases, where it has been necessary to temporize heretofore, you can now do permanent work.

*Suitable cavities for porcelain.* To enumerate the different cavities in which porcelain can be used, would be to practically name all the cavities that occur, from the central incisors back to and including the upper and lower third molars. I will only cite a few cases and conditions in which porcelain will do a great deal better service than gold.

In those extensive cavities where decay has encroached so close upon the pulp that death of the pulp would almost surely follow if filled with a metallic filling, although you have lined the cavity with the best non-conductor you know of, porcelain will give you almost absolute security, the pulp will remain alive and the tooth comfortable. Clinical experience has taught us that porcelain is the best non-conductor of thermal changes, and practically restores the tooth to as nearly a normal condition as though decay had never occurred.

Cavities on the buccal surfaces of molars and bicuspids at the gum margins, which are sensitive to anything hot or cold taken into the mouth, and cavities on the labial surfaces of the anterior teeth that are sensitive to the drawing in of a cold breath when filled with gold, become insensitive when the gold is replaced with a porcelain inlay.

Accidents to the young that result in the breaking off of a tooth, even to the extent of half the length of the crown, where it is desirable to retain the pulp alive on account of the incomplete development of the root, can be restored to full contour and usefulness with a porcelain tip and the pulp will remain alive.

Cavities from which the best inserted gold fillings are being constantly bitten out by force of mastication, can be filled with inlays that will stand all the stress that teeth should be subjected to.

*How porcelain should be manipulated, and why.* I expect to show you cavity formation and my method of burnishing a matrix at the clinic so will not take your time to describe them here. High fusing bodies are the best in every feature of inlay work. Low fusing bodies, i. e., bodies that fuse below the melting point of gold, do not produce the translucent natural-looking inlays that you can make with high-fusing bodies.

For illustration I will describe the handling of the different bodies in making an inlay for an approximal cavity in a central incisor extending from the cervical or gum margin to and including a third or half of the cutting edge. You select some one of the several good bodies that are on the market to build what I call the foundation of the inlay ; I use Close's body, or Brewster's Foundation : have it finely ground as it will pack more solidly, carve better, and shrink less than coarser bodies. Build it into the matrix little by little, jarring it down well to make it solid ; build out the corner in excess of what you expect the contour to be to allow for shrinkage ; when sufficiently dry that you can carve it, begin to carve and shape it, so that you will complete this work before it is dry enough to crumble ; carve the lingual surface right down to the contour the matrix gives you, then carve away what would be the labial half of the inlay, carve it away right up to the matrix so that the room for laying on your colors will be the same at the edge of the tooth as any other part of the inlay. Then bake, and if you have estimated your shrinkage correctly you have an inlay for the lingual half of the cavity only; if shrinkage has been more than expected build on more of the foundation and bake again; you are now ready to build on the colors you want the inlay to be. You can vary the shading from the neck of the tooth to its cutting edge at pleasure. I never mix two or more bodies together to vary the shade of one, but depend on the thickness of the layer to give me the shade I want; you can get any shade of a given color by the thickness of the layer of enamel you use. I can best illustrate this by showing the effect of holding a sheet of colored glass to the light; you get a certain shade of that color now; place another sheet back of it and you get a deeper shade of the same color and by adding more sheets of glass you get a still deeper shade of the same color. It is the same in using bodies; you can get any shade of a color by the thickness of the layer you use. Build your colors on in layers and bake each layer as you go along. When you have built your colors to almost full contour cover the whole inlay with a neutral color that will allow the underlying colors to reflect through; in this way you will get a translucent effect, and it is the only way that the translucency so desirable can be obtained. In the tooth you are trying to match, the colors are all in the dentin and reflect through the enamel. The enamel of all teeth is practically the same color, the different shades come from the difference in the dentin.

All inlays whether for restoring contours, or for simple saucer-shape cavities, I build up in layers, and never less than three layers; foundation, color and enamel. An inlay built up in layers accomplishes three objects: first, a natural translucent-looking inlay; second, if built of three or more layers of different bodies it will break up the absorption of light, so that from whatever angle or point of view you look at it, it will look practically the same. An inlay built all of one body or mixture will absorb light only from one direction, and viewed from one point will look right, but from the opposite point of view will show as differently as black and white. An inlay built in layers will come very near imitating nature's method of building up a tooth and by breaking up the direct absorption and refraction of light rays, will come very nearly looking the same from all points of view; third, you overcome that great bugbear of most inlay workers, the cement showing through after the inlay is set. An inlay built up in layers will prevent the reflection of the cement through from underneath. You will often hear operators say they had a splendid color before the inlay was set, but after it was set the cement killed it entirely. That was because the inlay was baked of one body only and the cement could reflect through from underneath as easily as the light was absorbed only in one direction from above.

The three points I claim therefore for this method; are, translucency, avoidance of shadow and prevention of cement reflection from underneath.

This brings us up to the method of selecting the colors for an inlay. I want to bring before you a new principle in color selecting. I will quote in part from a paper I read before the Iowa State meeting last month:

"It is the general practice to select the color as one selects a facing for a crown, by taking the shade guide furnished with every outfit of bodies and selecting the color that seems to be a match for the tooth. In selecting a facing general effect is aimed at and the selection is one that harmonizes with the adjoining teeth. A person might select a facing, that, placed between two natural teeth could not be detected, but if a corner of that facing was joined to the corner of either adjoining tooth it would not match at all, because each tooth has different underlying colors. It is underlying colors you want to look for. It will surprise you when you look at a tooth in this way what a different lot of colors you will see, and it is just in proportion as you are able to see these underlying colors and are then able to reproduce them in your inlay, that you will be successful. It will surprise you when you look in this way at what you would say was a typical light yellow tooth, to see that there is either a gray or blue tinge, or both, down inside that tooth. You want those same colors in your inlay or it will not be a perfect match. Now I use the shade guide not to get a match for the tooth, but to help me in looking for the underlying colors and the degree of those colors."

If you see yellow, brown, blue, gray, or all of them, hold the shade guide to the tooth and determine the strength of these colors, also the order in which you will use them to give you the effect you want. Make a memorandum of this; for instance, you have a typical light yellow tooth you are making an inlay for; the cavity extends from neck to cutting edge, involving at the cutting edge a third (more or less) of the width of the tooth; Yellow No. 2, on the Brewster shade guide looks to be a perfect match for the tooth, but at the neck you find the yellow a little deeper, with a grayish tinge through the center third of the tooth and a bluish tinge toward the cutting edge; now you will find on looking closer that the blue seems to reflect through a gray and that there is very little yellow in this part of the tooth. My memorandum would read thus: Patient A; foundation, cervical, No. 3 yellow; center, No. 6 gray; cutting edge, No. 9 blue under No. 5 gray; over all, No. 2 yellow with No. 11 for enamel. I would proceed to use them as follows: build my foundation as described; the yellow, gray and blue would then be put in their respective places and baked as one layer; have the three bodies properly moistened and arranged on the slab so that you know which is which; take as near as you can judge the proper amount of each and place at their respective places; then a slight draw of a knurled-handled spatula will draw the moisture to the surface and cause them to run together; cease instantly and you will have a perfect blending from one color to the other: if you continued to jarr you would get a mixture instead of blending; allow it to dry and bake. You will find shrinkage will necessitate the building on of more of these colors, and this time, as you want gray over the blue, you will only use No. 3 yellow and No. 5 gray; have the bodies ready and proceed as before. This will still further harmonize your colors because the yellow will extend still further down your inlay and modify the gray that was put on in the central portion, while the blue of the lower third modifies the gray, so that when they are all covered with the lighter yellow, you have such a harmonizing of colors that you can't tell it from nature's work itself.

If shrinkage causes any lack of contour and it most always does and is really to be desired, cover all with a layer of Brewster's X X body. This is a new product that Mr. Brewster has been working at for some time, and he brought me the first to test about two weeks ago. You can't say it is any particular color unless you call it enamel color; so far it seems to fill its mission perfectly. You now have a completed inlay that I believe comes the nearest to reproducing nature of any means or methods heretofore attempted. A still further artistic effect can be obtained by the use of what I call primary colors. They are as deep as black, Prussian blue, Van Dyck brown, burnt ocher, etc. They are high-fusing bodies and not paints, but they must be used as paints would be, by putting them on mixed with oil, for you can not put them on thin enough or smooth enough mixed with water as you do ordinary bodies.

These are a set of colors that were made for me by Mr. Brewster, of Chicago, and are meeting a long felt want. Their use is not confined to inlays but they can be used to artistically change facings and teeth for all kinds of artificial dentures. Mr. Brewster has now put them on the market in a very convenient form. They are a distinct, separate set from his regular set of bodies. With them you can reproduce that steel blue line you so often see just above the cutting edge, also the tobacco-stained teeth and the white and yellow mottled teeth. These effects have heretofore been impossible but with the primary colors they can be reproduced with life-like naturalness.

*Why is porcelain the most permanent of all the materials we use to-day?* There are several clinical facts in connection with inlays that are at present unaccounted for. First, there is practically never a re-occurrence of decay around a porcelain inlay; second, they stay in all cavities, under all conditions, better than gold fillings; third, when in contact with an adjoining tooth they are a protection and safe-guard against decay of said tooth.

To the first I will not try to give an answer, only to say it is an established fact, through the observation of all inlay workers that there is seldom or never any re-occurrence of decay around an inlay. This fact of itself is enough to place porcelain among the first as to permanency.

Second, here is where we have so many doubting Thomases; it seems almost impossible for a dentist to conceive of any other law of physics than the one he has been brought up on, that of a retentive form of cavity, and an interlocking form of filling material. It seems equally hard for the majority of inlay workers to get away from that same law.

In the April issue of one of our leading dental journals was printed a paper, that was read before two dental societies, in which the author gave his conception of a cavity formation that would be interlocking against lateral stress. There are others that are working along these same lines. This is a useless waste of time on the part of the operator and a needless infliction of pain upon the patient. Still others are baking into inlays platinum pins and loops for the purpose of retention. It has been a number of years since I abandoned this practice; this came about through having a tooth so sensitive that it was impossible for me to cut the pit for the pin to set in. It was a case in which an inlay was indicated and I made one without a pin in the cutting edge, trusting to luck that it would stay; it stayed just as well as any that had pins baked in. This set me to thinking and the result was that I abandoned all such means of retention. It took me longer to get away from the idea that I must undercut the cavity before setting the inlay and undercut the inlay as far as possible. It is about three years since I abandoned this practice, and I believe that clinical experience has taught me the true principle of inlay retention. It is close adaptation to all parts of the cavity, and setting the cement under pressure.

"It is exactly on the same principle as a joiner joins two pieces of wood; he prepares the surfaces to be joined so that they are in perfect adaptation to each other and placing glue on these surfaces, places them in a vice or clamps them together under as much pressure as he can until practically all the glue is squeezed out; and then he leaves it to harden, and the less glue there is the stronger the joint. This I believe is the true principle upon which inlays depend for their strength of retention. It was formerly my practice to score the reverse side of the inlay with a knife-edge carborundum wheel removing as much of the glaze as possible, but often inlays were of such size and shape as to make this extremely difficult. It has become my practice for some time to etch the reverse side of all inlays with hydrofluoric acid; this removes all the glaze leaving a roughened surface, but does not alter the close adaptation the inlay must have for the interior of the cavity as well as at the margins."

Third, when in contact with an adjoining tooth they are a protection and safeguard against decay of said tooth. I have always contended that an inlay should never be ground on any surface other than the occlusal surface after it is set; if any grinding is necessary it should be done before the matrix is removed and glazed again in the furnace.

The approximal surface of a gold filling or crown no matter how highly polished will retain fine particles of food settlement that are an exciting cause of decay. The natural enamel is easily attacked by the acids of fermentation and soon becomes roughened and hold increasing amounts of food settlement and decay follows. Glazed porcelain will not retain food deposit and is not affected by the acids of the mouth. Therefore the approximal space or contact point that one surface is restored with a porcelain inlay, lessens by more than half the liability of decay of the adjoining surface, while if the restoration had been by gold filling the liability would have been increased over the original conditions, for gold soon becomes tarnished and retains collections in excess of enamel. These, I believe, are sufficient reasons to establish the claim that porcelain is the most permanent of any filling material we have to restore lost tooth structure.

### DISCUSSION.

*Question.* Of what metal are your matrices made?

*Answer.* Platinum, of necessity, because all high fusing bodies fuse above the melting point of gold.

*Q.* Is it any more difficult to restore a lost part of a tooth where the color of the tooth has changed?

*A.* It is sometimes a little harder to match color.

*Q.* Do you allow for further change of color?

*A.* You can not do that in any of the work, you have to match the tooth in its present condition, your inlays may remain in for fifteen or twenty years, of course, the tooth will change in color in time, it is also possible that the inlay will change some. I have seen some of my inlays which have been in for ten years and they are looking just as well to-day as when they were put in.

Dr. Loeffler. The point that Dr. Reeves brought out in regard to close adaptation seems to be one which is of very general use in some of the arts. All joiners resort to this principle, and the main point in their work seems to be to get close adaptation. A day or two ago I was in a carriage factory where these principles were explained to me in a more definite way than ever before. The man said, "The closer the joint the better it is." The manner in which this was accomplished was by applying glue to the joint, placing two pieces in a vise and by means of this evenly distributed force less glue is required and the firmer the joint seems to be. I don't know whether there is a special explanation of that point or not, under the force the glue is perhaps driven into the material itself which is more or less porous. This could not be done in case of porcelain. I wonder whether Dr. Reeves could explain this fact in regard to porcelain surfaces. I would like to ask him whether fairly close adaptation with a large amount of cement would make it as strong as having adaptation very close and using very little cement.

*A.* Of course, in some works the glue is to a more or less extent forced into the pores of the substance, but there are various materials so dense that it is impossible to do so. I don't think that is the law of their holding. We have another illustration in the repairing of broken cut glass. The pieces after being placed together and glue applied, are forced tightly in contact and so close is this joint when completed, that it is practically impossible to see the fracture.

I presume if we could obtain the pressure necessary, the cement could be forced into the tubuli of the dentin as the case of the glue in some of the woods.

*Q.* Will Dr. Reeves give us an idea of the form of the matrix which he uses?

*A.* This will, of course, be brought out in my practical demonstration this afternoon, I might outline it, however.

In starting a matrix take a piece of platinum large enough to cover all margins and surfaces of the tooth to a considerable extent other than at the cervical surfaces. Place between the teeth, if approximal, and I will speak of this as being a typical illustration which applies to all cavities. Place this between the teeth and press into the cavity with wet cotton pellets, wet will pack easier than dry, and follow with other pellets until the matrix is fully pressed into the cavity. It should be forced into all portions of the cavity. Remove matrix and if at the cervical margin there are overlapping portions which hurt the gum trim away. Each time you can, you should anneal the platinum so as to make it work easier. After getting it thoroughly adapted to the cavity take a piece of tape pass up between teeth at one draw, draw so that the tape will pull in all directions. If you attempted to adapt one margin at a time you will get your buckle all in one portion, and it will be harder to burnish out the wrinkles than if you took a piece of tape and burnished in all directions as indicated. After doing this you can then complete the burnishing of the matrix into the cavity. This usually consists of four stages, those just given being the first two. In the third stage hold the matrix to the tooth as well as you can and where the platinum extends over the margin this can be done with the fingers, and then with burnishers burnish over all the margins. In doing this see that the pressure is equally distributed in all portions, you may possibly cause the matrix to spring away from the cavity at this time. It is just at this stage that a good many men try to introduce some method which will take away that spring, this is hard to do, it is impossible to do by any swaging method. If after you have practically completed burnishing of margins you take a rubber dam and draw down tight over the matrix and then go over this with your burnisher you will have a matrix which is as nearly perfect as possible.

*Q.* I would like to ask the Doctor what there is about porcelain inlays which makes it easier to prepare a cavity for them; is it not necessary to remove all decay?

*A.* Of a necessity you have to remove all decay, but in the use of any other material it is then necessary to cut retaining forms into the tooth to hold the filling in place. You can not even put in a cement filling without some retaining form. Retention forms are not necessary for porcelain inlays. And it is not the cement only which holds them in place, the principle of close adaptation is the reason why it stays in. A cement filling would not remain in the same cavity. Since it is not necessary to cut the under cuts into the tooth you have lessened the pain in preparing the cavity to a great extent. In this respect it is much easier to prepare a cavity.

DR. DAY. The Doctor speaks of a perfect adaptation. I have had some few years of experience and my observations have lead me to believe that there is no such thing as perfect adaptation by taking an impression or anything in that line. I would like to know how he holds these inlays in the cavity after having them prepared and in cementing in. He said the wood-worker holds his by means of a vise, how is he going to accomplish the same thing in the mouth? I have a simple way of my own, which is perhaps like nobody else. I have never been able to hold an inlay of gold or any other material into the cavity satisfactorily to myself without first making a preparatory crown or band, something that would hold it tightly in place until the cement had hardened. I have tried having patients holding it in with the fingers but they usually become tired before the cement will harden, and the inlay falls out.

*A.* In connection with the cavity preparation while you do not need any undercuts in any manner at all, you do want to have what might be termed a seat for the inlay. Be it ever so slight it amounts to a seat. This is accomplished by forming a kind of a triangular bottom any two angles being opposite each other makes it difficult to rock the filling out of place. It is an easy matter to wedge an approximal filling in place. This is done by an orange-wood wedge between the two teeth. After placing inlay into cavity see that all margins are equally in position. Then with a large wood wedge you can get all the force that is necessary, occasionally you will have to use two wedges, one at the cutting edge and one at the cervical but it is very seldom necessary. Cavities on the buccal and labial surfaces where it is impossible to wedge you can pass a couple of ligatures around the tooth previous to putting cement in, being careful of course to have them far enough out of the way so as not to interfere with the placing of the inlay. Everything must be ready and the cement mixed thick enough so as not to occupy more than ten or fifteen seconds in putting in place. If the ligature is not tight enough you can slip a wedge underneath and press the inlay into place as easy as if it were an approximal filling. It takes about fifteen minutes for expansion to take place in cement and this expansion is very necessary in the setting of inlays. Cements used which possess no expansibility are those where the inlay usually comes out. I leave the rubber dam on thirty minutes after putting the wedge in between. I then feel confident that the inlay is there to stay.

*Q.* You avoid the line of demarcation always?

*A.* So nearly that it will look like a check in the tooth, not one large enough to be called a crevice.

*Q.* What cement do you use?

*A.* Harvard Cement. I think that all inlay workers use it.

*Q.* When do you trim the matrix?

*A.* The first trimming is done only for the cervical margin. The next trimming is when the platinum is wrapped around the bell of the tooth, trim just where it will not hold into the cavity by lapping.

You need the platinum extending slightly over the edge of the cavity for two reasons. Rigidity of matrix which will avoid any danger of changing at margin while building up porcelain; and also in giving the contour to the inlay.

Dr. Moore. Dr. Reeves has described or spoken of the approximal cavities just a few minutes ago, does he mean an approximal cavity in an incisor or bicuspid? If so, I would like to ask him what he does in the large porcelain fillings between bicuspids? That is the location where I experienced considerable difficulty in this work some eighteen or twenty years ago. After removing the matrix on having it nicely adapted to cavity and when I built the coutour to the necessary size and went to slip the inlay in place the other tooth was in my way and I could not get the inlay in. It is not so bad with fillings in the incisor teeth, it is not necessary to make so large a contour and it is easier of access. The trouble with the bicuspid inlays is caused by either of two reasons: the inlay is too large in the anterior posterior direction, or it was too wide at the base. If I wanted to get in these locations it would be necessary to sacrifice a considerable amount of tooth substance which I would not think advisable.

*A.* In regard to that it is only a matter of space necessary to restore the tooth to its proper contour. It is just the same with a gold filling as with a porcelain.

Dr. Moore. The same principle does not apply in the making of a gold filling, that is put in piecemeal, each piece being carried to place, but the large mass of porcelain must of necessity be in one piece and the trouble arises in getting it into place.

*A.* If you have made the porcelain inlay to the ideal contour and have not room to set it, it is because you have not the space which should have been obtained at the first. A gold filling is not larger than the space you had there. You can set your inlay without any difficulty by preparing the tooth properly in the first place. I always separate whether for amalgam, gold or anything, where I wish to restore tooth to normal contour. Sometimes when you try the inlay in the tooth and it is too large to go down into place, you can grind the surface of inlay before taking off matrix until it will slip down but it is then necessary to put in the furnace and polish again. A roughened surface will always collect and hold particles of food.

Dr. Dorrence. I want to add a word to what has already been said. Reference has been made to joiners in making glue in making a joint. It would be a very green man that would trust to the glue for a perfect joint, the more glue the worse the joint and the less strength. I am not a joiner but know something about it. Most of the glue is forced from the joint and all that is left is simply a means of adaptation and exclusion of air. Those who have used inlays for any length of time and especially those who have tried to do good work and have had failures and had their inlays drop out have noticed that the cement is much denser in the cavity than any ordinary cement filling. You all know what a difficult matter it is to cut that thin hard line of cement from the cavity. The cement in these cases is rendered so much more dense that it protects the tooth thoroughly from recurrent decay.

*Q.*. I would like to ask the Doctor if he uses the rubber dam in all cases? I know it is proper to use the dam for setting the fillings, but do you use it in the preliminary stages?

*A.* I never use it until I am ready to set the inlay. I always use it for setting wherever I can get it on the tooth, and in cases that I can not, I attempt to get the mouth in a dry condition and keep it so, and as soon as the wedge of wood is in place or the ligature is tied around the tooth I cover the whole thing with melted parafine to protect it from moisture. It is not advisable to use a rubber dam in preparing cavity because a tooth will dry out and when you are ready to select color the tooth will be much lighter than normal, and in matching the inlay to the tooth in this condition, when the tooth resumes its natural color the inlay will be one or two shades lighter. It is always advisable to show the inlay to patients previous to being set. The inlay naturally looks better in the saliva and is then the same color as it will be later and the patient looks at it and forms that impression which will remain with him so far as that inlay is concerned. The first impression is always the best. As I said before I consider an inlay a success which will not be noticed in ordinary conversation, of course after once locating the inlay you will notice it ever afterwards. It is just like the puzzle picture in the paper, you may look a long time before you find it, but after once finding it you will always see it.

Dr. Loeffler. I believe there is a mistaken idea in regard to the fact that it is simply close adaptation that holds in the case of a joint. In the two pieces of wood or the glassware, or the adaptation of porcelain inlay to the tooth, close adaptation could be gotten and pieces held together by other materials which would not serve the purpose. If the joiner got perfect adaptation and used molasses or something else of that nature I am sure the pieces would not hold together. There is something in having a good form of glue. Close adaptation is not the point altogether. In reference to the remark of Dr. Dorrence "the less the glue the better the joint," we could draw from that remark that if we did not use any glue the better it would hold. I believe this is not the case.

Dr. Dorrence. I do not claim that if there was no glue present there would be a perfect joint. The glue is there for that purpose. In forcing this joint together it is very necessary that the air should be excluded. The same principle holding in the inlay of the tooth. The lasting qualities of a porcelain filling depend upon three things; first, the inlay should be correctly made; second, the right material should be used in setting same, and this should be a finely ground cement. By the use of a coarse cement and plenty of it, it would be no better than a porcelain filling. Third, setting it properly. A well made porcelain inlay is far ahead of any other filling.

Dr. Watson. It seems to me that it is not only the close adaptation of the inlay to the cavity but the close adaptation of the cement, the inlay and the tooth which is the principle that controls the making of a perfect inlay.

There were those who in the making of bands for teeth advocated making them loose so there would be plenty of room for the cement between the band and the tooth but we found these bands afterwards pulled off. Why? Because in placing this band on the tooth the cement was never under much pressure and the moisture easily penetrated between the cement and the tooth. It is possible with thin band material which is used in orthodontia, to adapt it so closely to the tooth that the developmental lines will show, and when we cement a band of that kind on to a tooth it is the rarest thing in the world that it comes loose. This is due to the cement being under pressure and also being closely adapted to the surface of the tooth and the band.

*Q.* I would like to ask Dr. Reeves if he has ever used hydraulic cement? There are some which harden under moisture.

*A.* I have tested them to a very limited extent. A few months since some Fellowship Cement was sent me, and I used it according to directions, allowing it to absorb a certain amount of water which they claim necessary, but of course the time is so short that it is impossible to say anything as to the success of these inlays. I would use this cement in cavities of teeth on which I could not put the rubber dam. I have a memorandum of these cases, and in a year will be able to report something definite.

Dr. Taft. This method of filling has been long enough used to demonstrate its value, and as was stated in the paper I think it is more durable in certain respects than any other material used. It does not disintegrate and there is no change in the filling and if well adapted to the cavity no decay takes place about it. That statement was more strongly made in this paper than I have ever heard it elsewhere. I have hardly ever seen a case in which recurrent decay took place about the inlay, unless there was a defect in the enamel or dentin. In regard to the words "perfect adaptation," so far as we are concerned, the word "perfect" must be used with discretion always. I don't know that we have anything which is perfect, we can simply approximate that condition. With the progress that is being made in the methods of manipulating porcelain I have no doubt that ere long it will be used by everybody and the possibility of decay will be precluded in a large proportion of cases.

This method will to a certain extent at least, take the place of gold, not altogether of course, but it will be much more used than it is now. Almost everybody thinks he can take a piece of platinum, adapt it to the cavity, build up a porcelain inlay and cement it into place. But there are as many fine points to be observed and practiced in the use of this filling as gold and perhaps more. We ought not to rest satisfied with coming here and hearing these things, every one who desires to keep up, must take it up seriously and in earnest. The majority seem to take it as mere by-play, and not with the same earnestness and effort put forth to make gold fillings. If we should spend a similar amount of time to that required in some institutions of learning, to acquire skill for filling teeth with other materials, we could make just as good porcelain fillings as Dr. Reeves perhaps. I hope for the day when it will supercede wholly and everlastingly amalgam, which should never be used again. In the clinics in our institutions, hundreds, yes I might say thousands of amalgam fillings are taken out, and not one filling in ten of this kind protects the tooth against recurrent decay. If this material shall be the one to wholly supplant this filling, that alone will make it desirable. In our institutions it should be taught, the same time and attention should be given to it as is given to other materials. Some twenty years ago I saw a porcelain filling put in by the late Dr. Goddard, of Louisville, Kentucky. It was in a superior central incisor and had been worn fifteen years without any decay about it. The inlay had been ground down from a piece of porcelain to fit the cavity. A great deal is needed in the way of improved appliances for this work; those who have taken hold of it more recently are the ones who have acomplished the best work.

Dr. Day: Dr. Taft has hit on the vital point, we have the appliances for doing this work. For those operators who have not the privilege of an electric current there is no furnace which is perfectly satisfactory for fusing the high-fusing bodies. I started eighteen or twenty years ago when the work first started, and was quite enthusiastic, but owing to there being no satisfactory furnace, and after a number of failures, I practically abandoned the work. I have since bought furnaces but still have the same difficulty. If there is any furnace which we can use besides the electric, I would like to know about it. This is the most desirable work in operative dentistry and should be generally adopted, but we have to have the means at hand to do the work.

*Q.* I would like to ask Dr. Reeves if he confines himself to Brewster's Body entirely?

Dr. Reeves: I do not know of any furnace outside of the electric. I use Close's, S. S. White's and

Brewster's bodies. I have used a number of others but abandoned them, but will not name, because there is no use discrediting any body. I use Brewster's bodies most extensively, I do not name this for advertising purposes, but simply that Brewster has been working with me for a number of years preparing bodies to meet certain wants. Personally I like to see the man who has stepped in where there was no demand for his body, and created a demand for it through its good qualities. All the credit should not be given to those manufacturers who just supply a long-felt want. Brewster's Body is as good as any on the market. There are others probably as good, but none better.

I commenced with using gas furnaces and of course had a good deal of trouble from the inlays gasing, but I would not give up my knowledge gained from the use of these furnaces for a good deal. The electric furnace is the best, but the person does learn some of the fundamental principles of porcelain work with the use of the gas or gasoline furnace. I do not advise any one to buy a gas furnace to gain this experience; he can obtain it second-handed from other persons who have gone through this hard experience.

